# Tools and Biomechanical Modeling Use in Legal Disputes: Some Case Studies

**DOI:** 10.3389/fbioe.2019.00429

**Published:** 2019-12-17

**Authors:** Francesco Durante

**Affiliations:** Department of Industrial and Information Engineering and Economics, University of L'Aquila, L'Aquila, Italy

**Keywords:** forensic biomechanics, movement analysis, free body diagram, multibody model, accident reconstruction

## Abstract

The paper presents some of the biomechanical tools available for the forensic bioengineering expert. The tools range from the simple three-dimensional modeling environment to represent the geometries up to the analytical models based on the free-body diagram and the multibody numerical models of rigid bodies. Through these tools the forensic bioengineering expert is able to solve complex problems by providing quantitative results based on a scientific approach. In this work three case studies, representing real cases that were treated in court, are presented. They relate to accidents which occurred in different contexts. The first relates to an accident in a filament factory where a worker remained with her body stuck in the production line, the second the hit of a pedestrian, and the last concerning a worker who fell from a wall on a construction site. It is shown that the approach to modeling may not always be necessarily complex. It was possible to solve the first case with a simple three-dimensional geometric model that clearly highlighted the development of the facts. In the second case it was possible to set up a simple analytical model based on a free body diagram to search for the relationship between the forces developed on the invested leg, demonstrating the relationship between the accident and the injuries reported. The third case, with the need for more complex modeling, was instead treated with a kinematic and dynamic multibody model which allowed the dynamics of the accident to be traced, starting from the final position in which the victim was found. In each case, the competence of the forensic bioengineering expert was crucial in identifying the correct modeling for the case in question, with the choice of the right data, in order to arrive at reliable quantitative results.

## Introduction

In recent times bioengineering experts are increasingly involved in forensic disputes given the complexity of devices with which human beings are in contact. In forensic bioengineering, a major part is carried out by using biomechanics.

Typically, in the event of accidents, a reconstruction of the dynamics may be requested or, for example, the response of injuries to an alleged accident at work may be requested. Sometimes the dynamics are known from the qualitative point of view and the expert is required to carry out a quantitative evaluation. This is the case of road accidents or accidents at work where an authority has emerged for the findings of the case. From the collected data it is beyond doubt that an accident occurred, but it is necessary to carry out an accurate dynamic reconstruction to highlight the behaviors and responsibilities of the person/persons involved. The expert must be able, through kinematic, dynamic, or structural modeling, to reconstruct the movement, over time, of bodies such as, for example, those of a car in relation to the impact with the body of a pedestrian or a motorcyclist. In other cases, although an accident has occurred, there is no certainty that some of the complained of injuries occurred in relation with the accident; or there are injuries, but there is no certainty that these are related to certain working conditions. In this case, the expert may be required to assess the compatibility of the injuries or damage claimed with the accident, or with the given working conditions.

From the above, the problems facing the biomechanical expert are very varied in context and for issues to be resolved, there is no single tool or approach that can be considered valid for every problem. In some areas, in which the problems are homogeneous, have by now definite approaches, such as in the case of road accidents, but in other areas, such as in the case of accidents at work or at home, problems can differ greatly from one another and must be dealt with case by case using instruments considered on a case by case basis.

The tools available to the forensic bioengineer, in the context of problems attributable to mechanical traumas, range from simple three-dimensional geometric models to analytical dynamic models based on free body diagram modeling, or modeling based on the principle of momentum and on the principle of conservation of energy, to kinematic and dynamic numerical models of rigid body systems, and to finite element models for structural investigation.

The 3D modeling of the bodies and environments in which the accident occurred is of great importance given the ability to report a large quantity of information all in the same space, and place it in a reciprocal relationship which helps to exclude the possibilities that manifest inconsistency.

In some cases, accurate three-dimensional modeling can be enough to highlight elements of the dynamics of the accident which are useful in a forensic dispute.

Dubik et al. (2016) demonstrated that three-dimensional modeling by laser scanners can greatly assist the bloodstain pattern analyst with the scene examination and provide an accurate area of origin determination, while in Liscio et al. (2019), the authors investigated the accuracy and reproducibility of the bullet trajectory tools in a software package based on a three-dimensional model provided by a laser scanner.

Some cases may need to be traced back to the forces exchanged between the bodies that have come into contact and which may have caused injury, and building a dynamic model based on a free body diagram could be sufficient. The solution is possible analytically. With this type of modeling it is possible to treat static cases, or dynamic cases with few bodies involved.

In other cases, a complete kinematic and dynamic reconstruction is required in which the movements, over time, of bodies entering traumatic contact are fully described. Although in some specific cases, like the reconstruction of road accidents, the models are consolidated (Brach and Matthew Brach, 1987; Struble, 2013) and application software is available on the market. However, for accidents such as the cases of investment of pedestrians or collision between a motor vehicle with a motorcycle and the driver, or in all other cases in which anthropomorphic elements are present, reliable modeling is much more complex. When the anthropomorphic element is present, the principles of mechanics can be easily applied only if it can be treated as a material point or as a single rigid body. For example, the speed of impact of a motorcyclist against an obstacle that has unseated him must be determined, or the motion after impact of a pedestrian being hit by a car has to be determined. In these cases, it is sufficient to consider a ballistic model that links the range of a projectile with the initial speed and the initial angle to the horizontal, with the human body considered as a material point concentrated at its center of gravity. While the vehicle can almost always be considered as a rigid body, not so much can be done for the anthropomorphic subject that, instead, in certain cases, must be considered in all its complexity as an open kinematic chain with multiple branches constituted by numerous rigid bodies which are mutually connected, and which interact between them. If, for example, in a car vs. motorcycle accident, the exact post-impact dynamics of the motorcyclist must be reconstructed to verify the hypotheses before impact, it is necessary to use complex models that cannot be solved analytically. To this end, over the last few decades, numerical calculation software has been implemented which make it possible to calculate the evolution of complex multibody systems. These tools developed in the field of scientific research, based on industrial design methods (Ahmad and Himmler, 2001; Sid Wang, 2001) and on biomedical research and design (Reinbolt et al., 2011), enable the construction of 3D models of mechanical systems, each constituted by several bodies (Panero et al., 2020). By using this software it is possible to simulate the motion/interactions between the different parts. The systems in question are known as multibody systems and all the bodies are treated as non-deformable bodies. It should be kept in mind that there is a very broad class of problems that can be treated with this hypothesis. The software makes all the data calculated during the simulation available to document all the kinematic and dynamic parameters of interest (Raparelli et al., 2007; Koceska et al., 2009, 2013). Furthermore, some cases of a structural type involving mechanical stress or vibrational behavior can also be considered because certain software uses Finite Element Modeling inside multibody environment, although in a linear context. By means of multibody models, Schulz et al. (2008) investigated planar and three-dimensional simulations of an anthropomorphic test dummy falling from a bed and compared them with a common estimation method. Commonly, similar investigations have been carried out on an experimental basis (Bowers et al., 2008).

Since it is difficult to artificially generate the physiologically correct anthropomorphic movement, motion acquisition systems are used to import it into multibody models (Sun et al., 2018). As for 3D modeling, procedures are available for the construction of three-dimensional models by the use of laser scanners (Koceski et al., 2009) or photogrammetry.

The purpose of this work is to show the use of some of the tools presented above, with reference to three real cases which were dealt with in court. The cases are addressed with different context-dependent approaches. The critical aspects of each case are highlighted, and it is shown which is the most suitable tool for use to face the problem and to reach reliable quantitative results.

## Materials and Methods

In the following, three cases are presented in which, thanks to an approach based on quantitative methods and on the previous described tools, a forensic bioengineering expert succeeded in legal matters to assert objective and conclusive evidence that led to the court's decision.

**Figure 1:**
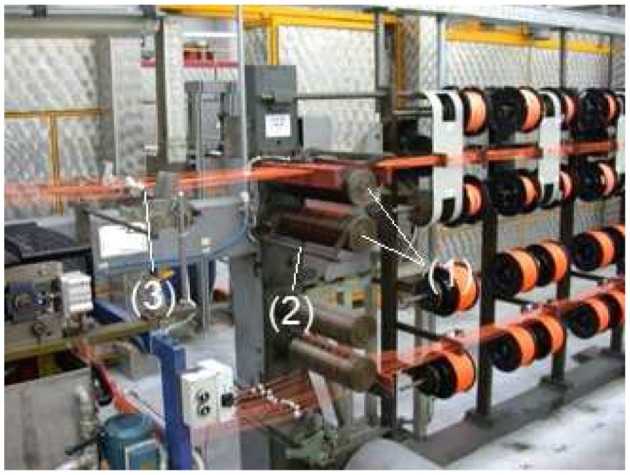
The working environment and the production line. It is possible to see the motorized rollers (1), the articulated guide (2), and the wire guide plates (3).

The first case concerns an accident at work. On a production line of threads for fabrics, there is a section in which different threads are spun together and then sent to the finishing oven. At the end of the section, before entering the oven, the wires are routed by motorized rollers that drag the wire and regulate the synchronicity of all the parts involved in the process. The operator must check that the process takes place in compliance with the production parameters, such as correct routing from the various packages to the wound wire, and tension of the wound wire entering the oven, etc. For the routing of the wire there are articulated guides on which the operator can intervene. If the guides are raised, the process stops by blocking the motorized rollers. In this case the accident occurred because the operator's arm was caught between two motorized rollers which caused her injuries. A dispute arose in which the operator requested compensation for damages, while the company claimed responsibility for the incident against the worker. An expert was appointed by the court who expressed that the plant could be considered in compliance with the law and that substantially, the responsibility was attributed to the worker who had not acted promptly on the articulated guides to stop the motorized rollers. The expert described the sector of the machine where the accident occurred. He described the superimposed motor cylinders in which the worker came to be in contact with the forearm (Figure 1). The expert also described the devices called articulated guides which, in the event of detachment from the surface of the motor cylinders, determine the arrest of the cylinders themselves. The operation and position relative to the drive rollers were also described. He emphasized that the operational purpose of the aforementioned articulated guides is strictly connected to the production cycle. The expert also described other devices, called wire guide plates, located at a distance of about 75 cm from the drive rollers, which in the event of accidental or operational movement (since they are connected to the production cycle) block the movement of the motor rollers. The case was solved by the use of a three-dimensional geometric model of the work environment together with the accurate modeling, regarding the dimensions and proportions, of a mannequin resembling the worker. By using the 3D model, a comparison of the mobility of the worker with her arm blocked, and the possibility to avoid the accident, was put into evidence. The results are presented in section Accident on the Finishing Oven Line.

**Figure 2:**
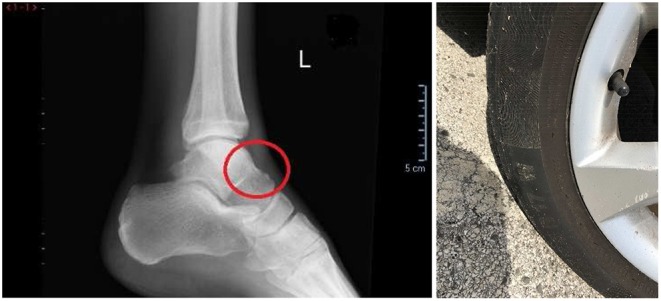
**(Left)** The fracture on the talus neck in the red circle. **(Right)** The wheel to be investigated whose size is 215/55/17.

The second case is about a traffic accident in which a car hit a pedestrian. Due to the hit the pedestrian reported the fracture of the left astragal, various excoriations on the left side of the body, and blunt trauma of the spine to the cervical level. The experts were asked to verify the compatibility between the accident and the documented injuries. The reconstruction of the accident proved as compatible the fact that the pedestrian was hit on the left foot, by the car's right front wheel, when the pedestrian had his left foot at the end of the stance phase and was about to begin the swing phase of his ambulation. Typically, the neck of the talus fractures when there is a forced dorsiflexion that occurs when the tibia is pushed, by external forces, toward the tip of the foot. Cases have occurred, for example, when falling from a high wall and landing with a crouching movement, or when in a car accident with an impact against an obstacle, the driver is pushed forward against the pedal board. The first cases of fractures of the neck of the talus date back to the first accidents of aircraft that crashed and the above-described mechanism was activated upon contact with the ground (Shamrock and Byerly, 2019). In the present case, the talus was fractured at the neck (Figure 2, left). The hypothesis that the fracture was due to the hit by the wheel, had to be investigated. Figure 2, on the right, shows the wheel which has size 215/55/17. This case was solved by the use of an analytical model, based on a free body diagram, which was able to relate the force acting on the calf to the force acting on the talus neck, and by computing the stress to which the talus was subjected. The results are presented in section Investment of a Pedestrian.

The third case concerns an accident on a construction site. A man was walking on a road along the edge of which there was a low wall, beyond which there was a drop of about 3 m. Near the wall there was a pipe which was necessary for the work. The man suddenly fell over the wall. In this case it is necessary to reconstruct the dynamics in order to understand how the man happened to fall over the wall. Did the man trip over the pipe? Did the man move forward toward the wall? Or did he move backwards and stumble? Following the incident, the man hit his head on the ground and did not remember, and there were no witnesses. Those who helped the man after the fall did not see the dynamics. The only data available is that the victim was found on the ground in a position approximately perpendicular to the wall, with his feet near the wall and his head away from the wall with the face upwards. To solve the case, whose results are presented in section Accident on Construction Site, a simplified analytical model was considered at first, but this proved to be unreliable. Then a multi-body model approach was used, which was solved numerically by using commercial software.

## Results

### Accident on the Finishing Oven Line

From what the expert wrote, the consultant for the worker derived that the operator is not required to consider the articulated guides as useful devices to stop the movement of the machine in conditions of emergency. Also, as for the wire guide plates, it is useful here to point out that the operator is not required to consider them as useful devices to stop the movement of the machine in emergency conditions.

The consultant for the worker carried out a three-dimensional modeling to better gather all the available elements, and to be able to carry out the necessary assessments as precisely as possible. He prepared an accurate three-dimensional reconstruction of the accident's environment. After a precise survey, he reported, and positioned in the CAD environment, all the most important parts of the spinning section before the finishing oven with all the elements that intervened in the accident, namely: the frames supporting the functional parts of the plant, the yarn carrying bobbins, the motorized rollers, and the articulated guides. He also built a dummy of the same size as the worker. The worker, whose height is 1.6 m, had proportions considered according to the model by Drillis and Contini (1966). In Table 1, the parameters used for the geometry of the mannequin resembling the worker are presented. Through the documents in the medical records, and based on the evidence of the accident, the worker's consultant positioned the dummy in the plant as the worker was at the time of the accident.

**Table 1:** Dimensions of the anthropomorphic model used for the investigation.

**Body part**	**% of body height**	**Height [m]**
Head + neck + trunk	13 + 5.2 + 28.8 = 47	0.208 + 0.083 + 0.461 = 0.752
L. upper arm	18.6	0.298
L. forearm + hand	14.6 + 10.8 = 25.4	0.23 + 0.173 = 0.407
L. thigh	24.5	0.392
L. lower leg + foot	24.6 + 3.9 = 28.5	0.397 + 0.062 = 0.459
R. upper arm	18.6	0.298
R. forearm + hand	14.6 + 10.8 = 25.4	0.234 + 0.173 = 0.407
R. thigh	24.5	0.392
R. lower leg + foot	24.6 + 3.9 = 28.5	0.397 + 0.062 = 0.459
Shoulder width	25.9	0.414
Hip bone width	19.1	0.301
Foot length	15.2	0.243
Foot breadth	5.5	0.088

*All the dimensions are related to a height of 1.60 m*.

**Figure 3:**
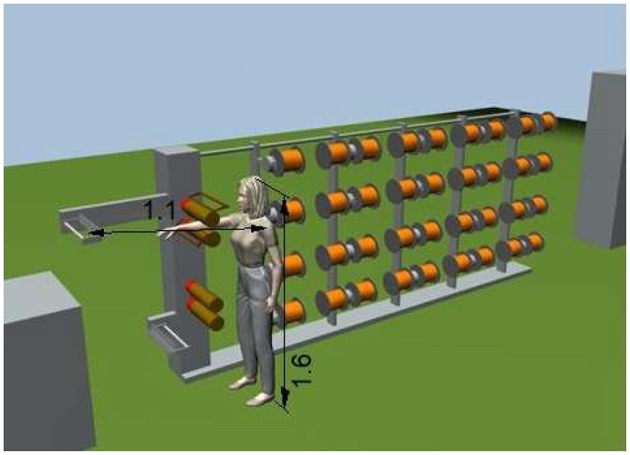
The three-dimensional geometric model of the detailed environment and of the mannequin representing the worker together with the parameters that permitted the technical assessments to be made.

The result was the three-dimensional geometrical model with the anthropomorphic element shown in Figure 3. The consultant pointed out the fact that, during the accident event, apart from the panic situation in which the worker found herself and which certainly prevented a lucid thought on what to do, behavior to which a worker is not used to, it is the equipment that must avoid emergency situations. Even while admitting that the worker had the time and the cool head to act on the articulated guides or on the guide-wire plates, the situation that was created prevented her from acting on these devices. In fact, given the small stature of the worker, and given the position reached by her arm in contrast with the upper motor rollers, it can be immediately deduced that she would never have been able to act on the aforesaid devices because they were not within her reach. The articulated guide was blocked by the arm itself which, when stuck between the rollers, kept it close to the lower roller. Moreover, given that the arrangement of the axis of rotation of the right elbow was almost horizontal, the right shoulder was practically blocked and therefore, also the bust. Hence, the wire guide plates, that are 1.1 m away from the left shoulder of the worker, were out of the range of the left arm which was free and the only one able to move, and whose length was 0.705 m up to the tips of the fingers. Figure 3 shows the three-dimensional representations of the work environment, reconstructed with the production line and a female figure, showing the left arm blocked from acting on the articulated guide and compared with the distance of the wire guide plates, which are out of its reach.

### Investment of a Pedestrian

**Figure 4:**
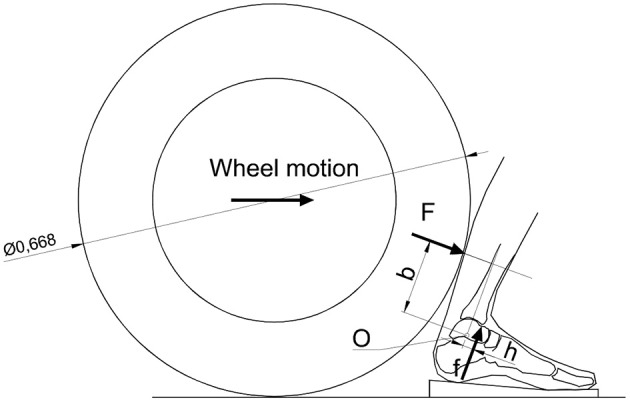
Possible configuration in which the wheel may have come into contact with the calf. It results in a multiplication of the force from the calf to the talus neck equal to the ratio of the arms with which the forces act.

In this case, the reconstruction carried out by the experts shows the compatibility of the action on the calf by the wheel that led to the kinematic block of the tibia on the neck of the talus. However, the medical expert considered this mechanism impossible since the medical documents did not report any damage to the calf. The expert essentially declared that the fracture in the incident was impossible, since it is not possible that an action that causes a rupture in another area of the body, that is further away from the point of application of the force, leaves no sign where the action has been directly applied. Although this discourse may have a general validity from the dialectical point of view, it is clear that it is only based on qualitative reasoning, which leaves out any quantitative evaluation necessary for it to leave the field of supposition nd enter the field of scientific fact. In fact, through a simple biomechanical model, it was possible to demonstrate that the fracture was compatible with the accident, along with the absence of any damage to the calf. In detail, the talus-tibia mechanism was modeled considering the joint as a hinge centered on the curved center of the mating surface of the talus with the tibia. Considering the free body diagram of the tibia, it is possible to create a correlation between the force acting on the calf and the force acting on the talus (Figure 4). The wheel, whose size is 215/55/17, has a diameter of 668 mm. By the torque equilibrium of the tibia around the ankle hinge O, we have:

(1) $$\begin{array}{r} F\;b\;-\;f\;h=0 \end{array}$$

where:

$$\begin{array}{l} F=\text{force acting on the calf by the wheel}\\ f=\text{force acting at the interface between the tibia and talus}\\ b=\text{arm by which}F\text{acts with respect to the ankle hinge}\\ \;=0.129\text{m}\\ h=\text{arm by which }f\text{acts with respect to the ankle hinge}\\ \;=0.018\text{ m} \end{array}$$

By the above equation it was shown that, thanks to the present mechanism and given the force *F* acting on the calf, a force *f* = *F* × (*b/h*) acts on the talus neck, which is more than 7 times *F*.

Subsequently, the pressure on the calf was considered equal to that exerted on the buttocks when an individual is seated. This pressure is certainly unable to cause trauma to the calf. It was assumed that this pressure exerted by the wheel on the calf, acted on a square area of about 70 mm on each side. Considering that the surface of the seat of a person sitting on a chair can have a rectangular shape of dimensions equal to 100 × 200 mm, and considering the action on this surface, due to the trunk plus upper limbs plus head for a total weight of about 600 N, the pressure is equal to 0.03 MPa. If we consider this pressure applied to the calf surface assumed to be affected by the wheel of an area of 4,900 mm^2^, we get a force of about 150 N. To determine the force that could have acted on the astragalus, this force must be multiplied by the force amplification factor of the mechanism, equal to 7.17, which gives a force equal to 1,055 N. Considering that the surface with which the tibia acted on the talus may be equivalent to a rectangle with sides 5 × 12 mm, in the considered hypothesis, a pressure of: 1,055/(5 × 12) = 17.6 MPa acted on the astragalus, which could be sufficient to cause a fracture of the talus which was considered as strong as a vertebra (Leondes, 2009; Zanetti and Bignardi, 2009).

### Accident on Construction Site

In this case, if we want to proceed with an analytical modeling it is necessary to greatly simplify the behavior of the human body by approximating it to a rigid parallelepiped element. In this way it is possible to hypothesize a speed with which the body moved toward the wall and obtain the motion of falling to the ground after the impact with the wall. The problem must be divided into phases. In the first phase we consider the body that moves horizontally toward the wall with an initial speed which is the speed of the impact with the low wall. At this stage, the action of gravity has no effect given the constraint constituted by the road. After the impact with the wall the body starts to rotate forward. Considering the principle of conservation of angular momentum (2), it is possible to derive the linear velocity and the angular velocity that the body has after impact with the wall. This state represents the initial conditions for the second phase of the problem in which the body rotating forwards, falls downwards due to the force of gravity, according to a ballistic motion. The motion can be seen as being composed by the ballistic motion of the body, whose mass is concentrated at its center of mass, with an initial horizontal speed equal to the speed immediately after the impact, plus a rotational motion around the center of mass with an angular speed assumed after the impact. In the case in question, the parallelepiped-shaped body is considered to have a mass of 75 kg with a height of 1.75 m, and with a horizontal translational speed of 0.5 m/s. The point of impact of the body is considered to be at the lower end of the body.

The equation that governs the first phase of the problem is:

(2) $$\begin{array}{r} mv_{i}b=I\text{ω}+mv_{f}b \end{array}$$

where:

$$\begin{array}{l} \;m=\text{mass of the body }=\;75\text{kg}\\ {\;v}_{i}=\text{velocity of the body center of mass before impact}\\ {\;v}_{f}=\text{velocity of the body center of mass after impact}\\ \;e=\text{coefficient of restitution}\\ \;\omega =\text{angular velocity of the body after impact}\\ \;b=\text{distance between the body center of mass and point of}\\ \text{ impact P }=\;0.875\text{m}\\ \;I=\text{moment of inertia of the body with respect to center of}\\ \text{ mass }=\;ml^{2}/12\\ \;l=\text{height of the parallelepiped shaped body }=\;1.75\text{m} \end{array}$$

As for ω it can be given by:

(3) $$\begin{array}{r} \omega =\frac{v_{f}+v_{Pf}}{b} \end{array}$$

where:

$$\begin{array}{l} v_{Pf}=\text{velocity of the impact point of the body after collision}. \end{array}$$

Since in the present case the point against which the impact takes place is stationary we have for the coefficient of restitution *e*:

(4) $$\begin{array}{r} e=\frac{v_{Pf}}{v_{Pi}} \end{array}$$

and since the motion before impact is translational we have *v*_*Pi*_ = *v*_*i*_.

By arranging the equations above we can obtain for *v*_*f*_:

(5) $$\begin{array}{r} v_{f}=\frac{v_{i}\left(mb^{2}-eI\right)}{mb^{2}+I} \end{array}$$

Thus, solving the first phase.

In the second phase the ballistic model of a projectile is used which starts from known initial conditions subject to the action of gravity. The model is expressed by the following equations:

(6) $$\begin{array}{r} s_{x}=v_{0x}t \end{array}$$

(7) $$\begin{array}{r} s_{y}=-\frac{1}{2}gt^{2}+s_{0y} \end{array}$$

where:

$$\begin{array}{l} v_{0x}=\text{initial horizontal speed of the body }=\;v_{f}\\ {\;s}_{x}=\text{abscissa of the body center of mass}\\ {\;s}_{y}=\text{ordinate of the body center of mass}\\ \;g=\text{gravity acceleration}\\ {\;s}_{0y}=\text{initial ordinate of the body center of mass }=\;0.875\text{m} \end{array}$$

Solving for the two equations we have:

(8) $$\begin{array}{r} s_{y}=-\frac{1}{2}\frac{gs_{x}^{2}}{v_{0x}^{2}}+s_{0y} \end{array}$$

**Figure 5:**
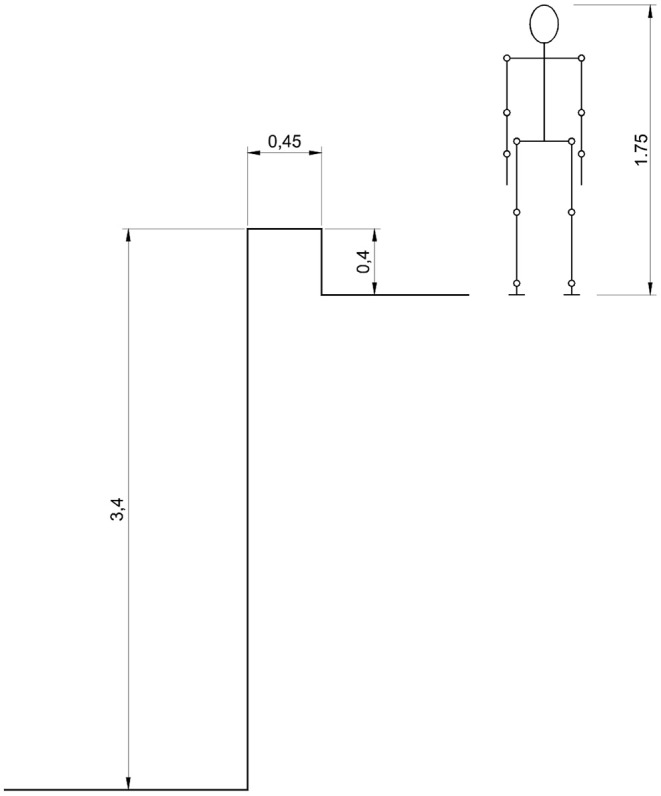
The mannequin considered with the height of 1.75 m compared with the wall from which the fall occurred.

Now for *e* = 0.5, the Equation (5) gives *v*_*f*_ = 0.31 m/s. In the real case the final value of *S*_*y*_ is *S*_*y*_ = −3 m, so that by Equation (8) it is possible to solve for *S*_*x*_, obtaining *S*_*x*_ = 0.28 m for a time, given by Equation (6), of *t* = 0.9 s. By Equation (3) it is possible to obtain ω = 0.643 rad/s. Now we can note that when the body impacts the ground after the fall, the ballistic range is 0.28 m at a time of 0.9 s, and the body is rotated forward about υ = 0.643 × 0.9 = 0.58 rad, which is about 33.2°. This is not consistent with what happened since 33.2° means the man almost landed on his feet, while in the accident, the man hit his head. This is because after the man stumbled into the wall, his body movement was constrained, rotating around the impact point in such a way the center of mass described an almost circular trajectory, before starting to fall freely according to the ballistic motion. In the model above we considered the ballistic motion to start just after the impact. Hence, the model should be modified in order to insert a third intermediate phase between the initial impact and the ballistic motion. But this complicates things due to the need of determining the exact configuration in which the falling phase started after the impact. Therefore, it is convenient to abandon this analytical model and to consider a multibody numerical model. This was implemented by using commercial software. A 9-segment mannequin was modeled together with the environment of the wall and the ground. The mannequin resembled the man to which the accident occurred, i.e., 1.75 m height and 75 kg of mass. As for the geometrical and mass proportions, the models by Drillis and Contini (1966) and of Pheasant (1986) were used, as in Durante et al. (2018). In Table 2, the segments considered for the model and their masses and dimensions are reported, and Figure 5 shows the scheme of the anthropomorphic model adopted, compared to the profile of the wall of the accident.

**Table 2:** Dimensions of the anthropomorphic model used for the investigation.

**Body part**	**% of body mass**	**Mass [kg]**	**% of body height**	**Height [m]**
Head+neck+trunk	6.2 + 2.2 + 50 = 58.4	4.65 + 1.65 + 37.5 = 43.8	13 + 5.2 + 28.8 = 47	0.228 + 0.091 + 0.504 = 0.823
L. upper arm	2.8	2.1	18.6	0.325
L. forearm + hand	1.7 + 0.6 = 2.3	1.28 + 0.45 = 1.73	14.6 + 10.8 = 25.4	0.255 + 0.189 = 0.444
L. thigh	10	7.5	24.5	0.429
L. lower leg + foot	4.3 + 1.4 = 5.7	3.22 + 1.05 = 4.27	24.6 + 3.9 = 28.5	0.430 + 0.068 = 0.498
R. upper arm	2.8	2.1	18.6	0.325
R. forearm + hand	1.7 + 0.6 = 2.3	1.28 + 0.45 = 1.73	14.6 + 10.8 = 25.4	0.255 + 0.189 = 0.444
R. thigh	10	7.5	24.5	0.429
R. lower leg + foot	4.3 + 1.4 = 5.7	3.22 + 1.05 = 4.27	24.6 + 3.9 = 28.5	0.430 + 0.068 = 0.498
Shoulder width			25.9	0.453
Hip bone width			19.1	0.334
Foot length			15.2	0.266
Foot breadth			5.5	0.096

*All the dimensions are related to a mass of 75 kg and a height of 1.75 m*.

**Figure 6:**
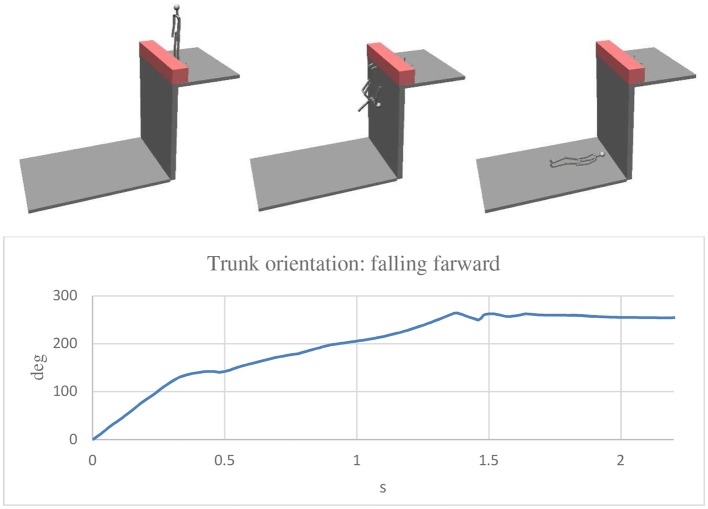
Simulation with the man walking with his face toward the wall. In the final position the trunk was rotated about +260° (forward) and the head was near the wall.

**Figure 7:**
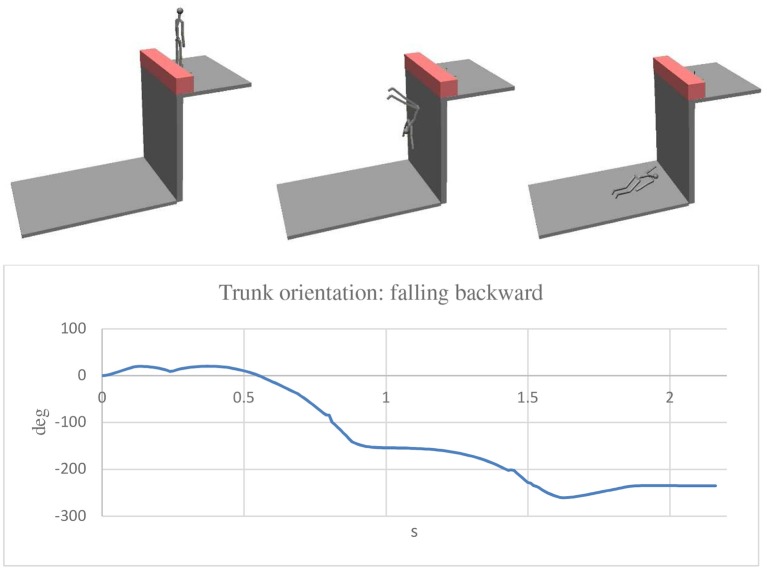
Simulation with the man walking with his back toward the wall. In the final position the trunk rotated about −235° (backward) and the head was near the wall.

**Figure 8:**
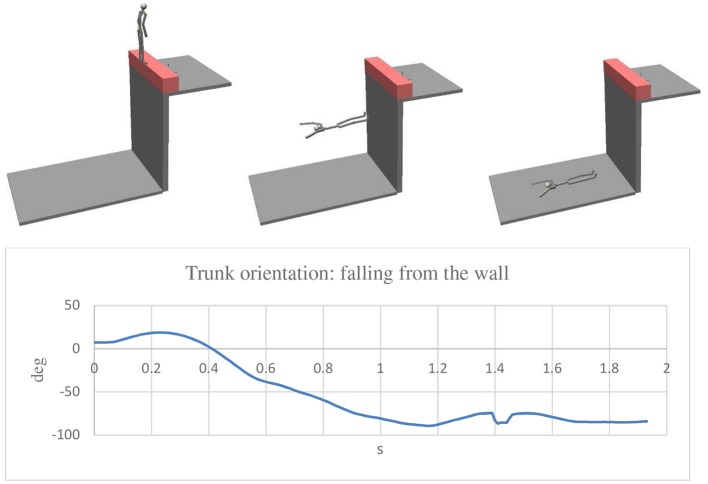
Simulation with the man walking on the wall with his back toward the void. In the final position the trunk rotated about −90° (backward) and the head was away from the wall: this is the situation compatible with the evidence.

For the assembly of the various parts of the mannequin, cylindrical constraints were used in all the joints except for shoulder joints for which spherical constraints were considered. In every joint, rotational springs and dampers were applied so as to resemble the muscular resistance of the human body. The constant of the rotational springs was 0.1 Nm/deg for all the joints, except for the hip bone-tight joints for which a value of 0.5 Nm/deg was used. As for the damper, a constant of 0.001 Nms/deg was considered for all the joints. A coefficient of restitution of 0.5 was used for the impact between the feet and the wall, and a value of 0.2 was considered for the impact of the body parts and the ground. As for the integration parameters, the Kutta—Merson method was adopted with an integration step of 0.01 s. By this model it was possible to analyse different conditions in a short time. A speed ranging from 0.5 to 2 m/s was considered as the velocity at which the impact of the man with the wall occurred. As previously mentioned, the problem to solve was to determine what were the real dynamics of the accident considering that no one saw the accident, and the victim did not remember anything. The Figures 6–8 show three simulations concerning three different initial conditions. There is the evolution of the trunk orientation, considered positive forwards and negative backwards, during the fall up to the static condition. The first (Figure 6) is related to the forwards movement of the man with his face toward the wall. The second (Figure 7) is related to the backwards movement of the man with his back toward the wall. The third (Figure 8) relates to a movement of the man over the wall with his back toward the void. The first result was that the speed of 0.5 m/s was not sufficient for falling in the first and second cases, for which a speed of the man of 2 m/s was necessary, while for the last case, a very small walking speed is sufficient. For the simulation a walking speed of 0.5 m/s was chosen. It can be seen that in the first case, the final trunk orientation was about 260°, which means at the end of the fall, the man had his face upwards with his head near the wall, but this was not the real case which occurred. In the second case, the final trunk orientation is about −235° which means the man had his face downwards with his head near the wall, so again, we have to conclude that this was not the real case which occurred. The condition corresponding to the real final state in which the man was found is related to the third hypothesized case, depicted in Figure 8, in which the orientation is about −90° with the head far from the wall.

## Discussion

In the first case the expert of the court stated the plant could be considered in compliance with the law and that substantially, the responsibility was attributed to the worker who had not acted promptly on the articulated guides to stop the motorized rollers. He stated this without any graphical documentation to support his statement. In this case it has been shown that an accurate static model, in terms of size and proportions, is sufficient to fully represent the situation of the accident and to show clearly and unequivocally how the facts took place. In particular, it was possible to demonstrate that the worker was not in a condition to avoid the accident from the time her arm was engaged by the motorized rollers. In fact, the worker could not act on the articulated guide to stop the process, neither could her free hand reach the guide wire blades, which were at 1.1 m from her left shoulder while her arm was only 0.705 m long. The evidence could not be obtained by the use of photographs in the phase of surveyor operations. Photographs could not be as effective as the three-dimensional CAD model was, since it was not possible to place the arm of a volunteer in the same position in which the arm of the worker was found in the accident. Even a two-dimensional representation, although with different views, would certainly not have had the communicative effectiveness of the three-dimensional environment. In this case the effective tool has been shown to be CAD software with photorealistic representation.

In the second case it was quantitatively demonstrated by a simple biomechanical analytical static model, based on a free body diagram, that a non-traumatic action on the calf, i.e., a pressure of 0.03 MPa on a 4,900 mm^2^ square area, can create a fracture at the neck of the talus due to a force amplification factor of more than 7, and a consequent applied stress on the talus neck of 17.6 MPa. In this case we were faced with the statement by a medical expert who had ruled out the possibility of a fracture without finding evidence on the calf. Under these conditions, the injury would not have been compatible with the accident in question. Thanks to the quantitative model by the biomechanical expert consultant, it was possible to overturn the doctor's statement and demonstrate the full compatibility of the lesion with the accident.

In the third case the problem required a solution that could not be easy solved with analytical modeling. It has been shown that with such simplifications as to allow an easy solution, i.e., considering the falling body as only one rigid body, the analytical model is not reliable. Hence, it was necessary to consider an anthropomorphic model with 9 segments for the solution, of which we proceeded numerically through a multibody approach. In this case the solution was more complex than in the first two cases. The determining element was to acquire the exact position in which the worker was found on the ground after the fall. Using the model, simulating the fall from the wall in different starting conditions and measuring the rotation performed by the bust during the evolution up to the ground, it was possible to identify the position that the body had before falling. In fact, the condition of finding the body with the face facing upwards and with the head away from the wall is compatible only with a fall from above the wall with the back facing the void with a rotation of the trunk equal to about −90°.

In conclusion, in forensic disputes in which a biomechanical expert could be involved, the questions can be very different depending on the case and there are no tools that can be used in any one situation. The different cases may require very different tools, of one form or another, depending on the type of data to be processed and the type of approach needed. Without wanting to claim to have classified all the necessary approaches, the three cases presented here show that sometimes a simple geometrical model may be sufficient, whereas at other times a dynamic model that can be solved analytically is necessary. Furthermore, there are times when we must proceed through more complex models that cannot be solved analytically, and for which numerical calculation is required.

## Data Availability Statement

The datasets generated for this study are available on request to the corresponding author.

## Author Contributions

The author confirms being the sole contributor of this work and has approved it for publication.

### Conflict of Interest

The author declares that the research was conducted in the absence of any commercial or financial relationships that could be construed as a potential conflict of interest.
